# Metabolic reprogramming related to whole-chromosome instability in models for Hürthle cell carcinoma

**DOI:** 10.1038/s41598-020-66599-1

**Published:** 2020-06-12

**Authors:** Ruben D. Addie, Sarantos Kostidis, Willem E. Corver, Jan Oosting, Sepideh Aminzadeh-Gohari, René G. Feichtinger, Barbara Kofler, Mehtap Derya Aydemirli, Martin Giera, Hans Morreau

**Affiliations:** 1 Department of Pathology, Leiden University Medical Center, Albinusdreef 2, 2333ZA Leiden, The Netherlands; 2 Center for Proteomics and Metabolomics, Leiden University Medical Center, Albinusdreef 2, 2333ZA Leiden, The Netherlands; 3 Research Program for Receptor Biochemistry and Tumor Metabolism, Department of Pediatrics, University Hospital of the Paracelsus Medical University, Müllner Hauptstraße 48, 5020 Salzburg, Austria

**Keywords:** Cancer metabolism, Thyroid cancer

## Abstract

Hürthle cell carcinoma (HCC) is a recurrent subtype of non-medullary thyroid cancer. HCC is characterized by profound whole-chromosome instability (w-CIN), resulting in a near-homozygous genome (NHG), a phenomenon recently attributed to reactive oxygen species (ROS) generated during mitosis by malfunctioning mitochondria. We studied shared metabolic traits during standard and glucose-depleted cell culture in thyroid cancer cell lines (TCCLs), with or without a NHG, using quantitative analysis of extra and intracellular metabolites and ROS production following inhibition of complex III with antimycin A. We found that the XTC.UC1 and FTC-236 cell lines (both NHG) are functionally impaired in complex I and produce significantly more superoxide radicals than SW579 and BHP 2–7 (non-NHG) after challenge with antimycin A. FTC-236 showed the lowest levels of glutathione and SOD2. XTC.UC1 and FTC-236 both exhibited reduced glycolytic activity and utilization of alternative sources to meet energy demands. Both cell lines also shared low levels of α-ketoglutarate and high levels of creatine, phosphocreatine, uridine diphosphate-*N*-acetylglucosamine, pyruvate and acetylcarnitine. Furthermore, the metabolism of XTC.UC1 was skewed towards the *de novo* synthesis of aspartate, an effect that persisted even in glucose-free media, pointing to reductive carboxylation. Our data suggests that metabolic reprogramming and a subtle balance between ROS generation and scavenging/conversion of intermediates may be involved in ROS-induced w-CIN in HCC and possibly also in rare cases of follicular thyroid cancer showing a NHG.

## Introduction

Non-medullary thyroid cancer (NMTC) is the most common endocrine malignancy^1^. Differentiated NMTC can be grossly subdivided in follicular and papillary variants. About 90–95% of differentiated NMTC can be cured by a combination of surgery and radioactive iodide treatment. Recurrence of differentiated NMTC is mainly seen in a subset of *BRAF*-mutated papillary thyroid cancers (PTC) and in Hürthle cell carcinomas (HCC). Although HCC explains only 3–10% of consecutive cases of differentiated NMTC, HCC accounts for up to 40% of recurrent cancers due to abrogation of sodium iodide (NaI) transport over the cellular membrane that impairs radioactive iodide therapy. HCC is now considered a distinct subtype of thyroid cancer^2^.

The characteristic oncocytic metaplasia found in HCC is caused by abnormal proliferation of inadequately functioning mitochondria^3^. Analysis of the mitochondrial DNA (mtDNA) in oncocytic lesions from different origins revealed frequent inactivating/disrupting variants, concentrating in the genes encoding subunits of complex I (CI) of the electron transport chain (ETC), thus suggesting an impact on metabolism^4^. However, other studies also identified numerous mtDNA variations in other types of NMTC^5,6^. Furthermore, a substantial fraction of HCCs entirely lack mtDNA variants^7–9^. Thus, mtDNA mutations cannot solely explain the oncocytic phenotype and might be a secondary effect due to mitochondrial hyperplasia caused by a coupling defect^10,11^. It has even been suggested that the oncocytic phenotype may be caused by mutations in oncogenes and/or tumour suppressor genes, with mtDNA variations accumulating as a late effect^12^.

In addition to an oncocytic phenotype, HCCs frequently show near-haploidy due to loss of complete chromosomes, but with retention of chromosomes 5, 7 and 12 in a heterozygous state^7,13,14^. Near-haploidy can be easily detected by multiparameter DNA content analysis^15^ and is frequently followed by endoreduplication or genome doubling of the entire near-haploid genome^7–9,13^, a common trait of solid tumours^16–18^. Endoreduplication of an entire near-haploid genome is sometimes referred to as ‘uniparental disomy’^19,20^. Thus, many HCCs show a ‘near-homozygous genome’ (NHG), a term that covers both a near-haploid genome status and the status after endoreduplication. We recently suggested that NHGs in HCC are the net result of a continuous process of whole-chromosome instability (w-CIN). w-CIN may be driven by factors such as increased intracellular production of reactive oxygen species (ROS) as a by-product of metabolic changes^21^ following the loss of CI functionality^22^. Interestingly, a recent study of renal oncocytomas found that recurrent CI loss was the earliest detected genetic alteration accompanied by altered metabolism and affected the production of the ROS scavenger glutathione^23^.

The aim of this study was to elucidate the metabolic features of thyroid cancer cell lines (TCCLs), focusing on ROS-driven w-CIN. To do this, we used metabolomics analysis^24^ in two TCCLs with NHG (XTC.UC1 derived from HCC and FTC-236 derived from conventional FTC) and two TCCLs without a NHG (SW579 and BHP 2–7). Furthermore, we performed spectrophotometric activity measurements of OXPHOS complexes in TCCLs. In addition we quantified superoxide and hydroxyl radical generation under normal culture conditions and after complex III (CIII) inhibition.

## Materials and Methods

### Cell lines and culture

The following cell lines were used: BHP 2–7 (derived from a primary papillary thyroid cancer)^25^, FTC-236, (derived from an FTC metastasis)^26^, SW579 (derived from a poorly differentiated thyroid carcinoma and deposited by A. Leibovitz) and XTC.UC1, the only model for HCC^27^. Cell lines were authenticated by a multiplex PCR (Cell ID GenePrint 10 system, Promega, Madison, WI), cultured for 20 passages and tested for mycoplasma at regular intervals (in-house PCR method). For detailed information, see Supplementary Table S1. Medium used: DMEM/F-12 (XTC.UC1, FTC-236 and BHP 2–7) or RPMI-1640 (SW579) supplemented with 10% foetal bovine serum (FBS) without antibiotics at 37 °C under 5% CO_2_. Standard RPMI-1640 media contains 3.3 µM reduced glutathione (GSH).

For metabolite analysis, cells were cultured for two weeks in DMEM/F-12 or RPMI-1640. Subsequently, ten replicates of each cell line were grown for 72 hours. Six and 24 hours before cell extraction, culture media were refreshed (controls) or replaced by glucose-free DMEM/F-12 or RPMI-1640 supplemented with 5 mM galactose (as a glucose substitute). In parallel, cell-free DMEM/F-12 or RPMI-1640 were also incubated for the same time periods and used as controls to calculate the consumption and production of metabolites.

The levels of ATP, ADP and AMP, as quantified from NMR spectra (see section “NMR spectroscopy and quantification of metabolites” below), were used to assess the adenylate energy charge (AEC) for each cell line. AEC values were 0.89 ± 0.02 for XTC.UC1, 0.90 ± 0.02 for FTC-236, 0.90 ± 0.01 for SW579 and 0.89 ± 0.01 for BHP 2–7.

### Metabolite extraction and NMR sample preparation

1. Extracellular metabolites: Culture medium (100 μL) was collected prior to harvesting and immediately mixed with two volumes of a pre-chilled (−28 °C) solution of methanol/chloroform/water (MeOH:CHCl_3_:H_2_O, 8.1:0.9:1 (v/v/v)). Mixtures were stored at −80 °C for at least 30 min, allowing protein precipitation. The mixtures were then centrifuged at 16000 × *g* for 10 min at 4 °C. Supernatants were dried under nitrogen flow and samples were stored at -80 °C until further processing.
2. Intracellular metabolites were extracted as follows. Cells were quickly washed with 5 mL MilliQ water at 37°C. Metabolic activity was immediately quenched by adding 15 mL liquid nitrogen to the petri dish and samples were stored on dry ice until further processing. The liquid N_2_ was discarded and intracellular metabolites were extracted at 4 °C with 1.5 mL pre-chilled (−28 °C) methanol/chloroform/water (MeOH:CHCl_3_:H_2_O, 8.1:0.9:1 (v/v/v) and detached and lysed with the aid of a cell scraper. The solutions were transferred to 2 mL Eppendorf tubes and centrifuged at 16000 × *g* for 20 min at 4 °C. Supernatants were dried under nitrogen flow.

Extracts were reconstituted in 250 μL of 0.15 M K_2_HPO_4_/KH_2_PO_4_ buffer (pH = 7.4) prepared with 99.9% deuterated water (D_2_O) containing 0.2 mM NaN_3_ and 0.4 mM trimethylsilylpropionic-d_4_ acid sodium salt (TSP-*d*_4_). From each sample, 190 µL was transferred to 3 mm NMR tubes using a Gilson 215 robot. Remaining volumes from each sample were pooled according to each condition and used for 2D NMR experiments.

### Cell counting

Cells were harvested using trypsin/EDTA and washed with HBSS. Cell counting was performed with a Muse cell analyser (Merck-Millipore) using the count and viability kit (product # MCH100102) according to the manufacturer’s instructions, after first calibrating the system with the Muse system check kit (product # MCH10010).

### NMR spectroscopy and quantification of metabolites

A detailed protocol for NMR spectroscopy and quantification of metabolites has been described previously^24^. Briefly, NMR data were recorded using a 14.1 T NMR spectrometer (600 MHz for ^1^H; Bruker Avance II) under standardized instrumental settings for all samples. Per sample, a 1D ^1^H spectrum was collected using the *noesygppr1d* pulse sequence, which is implemented in the standard library of the NMR manufacturer. The spectral data were phase and baseline corrected and referenced to TSP-*d*_4_ methyl protons at *δ* 0.00 ppm and subsequently imported into Chenomx NMR suit 8 (Chenomx Edmonton, Canada), allowing metabolite quantification. Metabolite assignment was based on the Bruker Bbiorefcode and Chenomx databases. The structures of all annotated metabolites were then confirmed in 2D NMR experiments of the pooled samples. The quantification of each metabolite was performed by integration of its proton peaks in the NMR spectrum using the deconvolution fitting algorithm of Chenomx NMR Suit. The calculated integrals were subsequently transformed to concentrations (mM), based on the known concentration of the internal standard TSP-*d*_4_ in each sample (0.05 mM). Finally, all concentrations were normalised to the cell population of each cell line. The quantities of ATP, ADP and AMP calculated from the NMR data were used to calculate the adenylate energy charge (AEC) of each sample. Uptake and release rates of metabolites by the cells, from and to the medium, respectively, were calculated using the following formula: *r* = (C_*spent*_ − C_*fresh*_) × V × 1000/A, where C_*spent*_ (mM) is the concentration of each metabolite as quantified in the culture media after 72 h of incubation and C_*fresh*_ (mM) is the concentration of the same metabolite in a parallel incubation of cell-free medium; V in litres is the volume of the culture medium (0.01 L was used in all cultures) and A is the area under the growth curve. The latter was calculated using the formula A = [(cells × d)/ln2] × (1-2^−t/d^), where cells represent the population of cells at 72 h, d is the doubling time and t is the time of incubation (i.e. 72 h). A positive value of *r* indicates release of a metabolite from the cell and a negative value indicates uptake in units of μmol/h/10^6^ cells. To represent uptake and release in the figures, the *r* values of each metabolite were divided by the mean *r* of that metabolite and reported as a relative change in the figure legend.

### Detection of superoxide and hydroxyl radical by quantitative flow cytometry

Superoxide and hydroxyl radicals were analysed semi-quantitatively by flow cytometry. Cells (250, 000) were treated and labelled according to the following scheme (see Supplementary Fig. S1). The following reagents were used: N-acetyl cysteine (Sigma-Aldrich, product # A9165), final concentration 2.5 mM; CellROX Green (Thermo Fisher Scientific, product # C10444), final concentration 5 µM; MitoSOX Red (Thermo Fisher Scientific, product # M36008), final concentration 2.5 µM; DAPI (4′,6-Diamidine-2′-phenylindole dihydrochloride, Sigma-Aldrich, product # D9542), final concentration 1 µM and antimycin A (AMA, Sigma-Aldrich, product # A8674). To find the optimal AMA concentration (range 1–800 µM for 2 hours), cell health (PrestoBlue assay), cell death (DAPI fluorescence) and ROS production were evaluated; 100 µM proved to be optimal (Supplementary Figs. S2 and S3).

Cells were analysed using an LSRII flow cytometer (20 mW 355 nm UV and a 20 mW 488 nm laser) after daily calibration. Fluorescence was collected using a 450/50 nm bandpass filter for detector B (DAPI fluorescence, dead cells), and a 610/20 bandpass filter (MitoSOX RED fluorescence) and a 530/30 nm bandpass filter (CellROX Green) for detectors D and F (blue 488 nm laser line), respectively. Cyto-Cal beads FC3MV (Distrilab, Leusden, Netherlands) were analysed allowing comparison of different measurements over time and expressing data as median equivalent fluorochrome (MEF). The experiment was repeated three times.

Data were analysed using WinList 8.0 (Verity Software House, Topsham, Maine). Ten thousand single live cell events were selected after doublet and dead cell discrimination using SSC (side light scatter)-W (pulse width) vs. SSC-H (pulse height), FSC (forward light scatter)-W vs. FSC-H, and FSC-A (pulse area) vs. DAPI-A dot plots, respectively. Median relative fluorescence intensities (MFI) were converted to MEF values. An ANOVA test was used to study statistical significance.

### Isolation of mitochondria and measurement of enzyme activity

Isolated mitochondria were used for enzymatic measurements. Enzyme activities of the OXPHOS complexes were measured as previously described^28,29^.

### Antibodies

Primary antibodies: anti-α-Tubulin Mouse, 1:50,000 (clone: DM1A, Cat. No. 14450282, eBioscience, San Diego, CA); anti-SOD2 Rabbit mAb, 1:1000 (Cell Signaling #13141). Secondary antibodies: Goat anti-Rabbit IRDye 800CW, 1:10000 (926–32211, LI-COR, Lincoln, NE); Goat anti-mouse IRDye 680LT, 1:10000 (926–68020 LI-COR, Lincoln, NE).

### Western blotting

Western blotting was performed as described previously^30^. Cells were washed with ice cold PBS and lysed with Hot-SDS buffer containing PhosSTOP (# 04906837001, Roche Diagnostics, Mannheim, Germany) and cOmplete (# 11697498001, Roche Diagnostics, Mannheim, Germany).

Protein concentrations were determined with the Bio-Rad DC Protein Assay (Bio-Rad Laboratories, Inc., USA). Ten µg lysates were mixed with 4x Laemmli Sample Buffer (# 1610747, Bio-Rad Laboratories, Inc., USA) containing βME, heated for 5 minutes at 100 °C and loaded onto a 1.5 mm 10% acrylamide gel (molecular weight markers: # 928–40000, LI-COR, Lincoln, NE). Electrophoresis was performed at 50 V. For blotting, the Bio-Rad semi-dry Trans-Blot Turbo Transfer System was used (Limit 25 V, constant 2.5 A, 15 minutes). Blots were then washed in Tris-buffered saline (TBS), blocked in Odyssey Blocking Buffer (# 927–50000, LI-COR, Lincoln, NE) at a 1:1 ratio with TBS for 1 hour, washed in TBS and incubated overnight at 4 °C with primary antibodies in TBS, 0.1% Tween-20, 5% BSA (Bovine Serum Albumin, Sigma-A9647, Sigma-Aldrich, St. Louis, MO, USA).

The next day, blots were washed in TBS and incubated for one hour with the secondary antibodies in TBS/0.1% Tween-20/5% BSA protected from light. After washing, blots were air dried and imaged at high-resolution with the Odyssey Infrared Imaging System (LI-COR, Lincoln, NE) using intensities 1.5 and 3.0 for the fluorescent colour channels red (700) and green (800), respectively. Images were analysed using Image Studio Lite Ver 5.2 software package (LI-COR, Lincoln, NE).

### Statistical analysis

Analysis and visualization of metabolomics data was performed in R statistical software (http://www.R-project.org/) using the packages *pheatmap, dplyr, ggplot, ggsci and RColorbrewer*, as well as GraphPad Prism version 8.0 (GraphPad software, San Diego, California, USA). Pairwise comparisons of metabolite levels between the cell lines were performed using the Mann Whitney U test and p-values were corrected using the False Discovery Rate method of Benjamini-Hochberg (BH). Cluster analysis of cell lines and metabolites was performed in R using the *factoextra* package, with Spearman correlation analysis.

## Results

### Citrate synthase and OXPHOS enzyme activities

**Figure 1:**
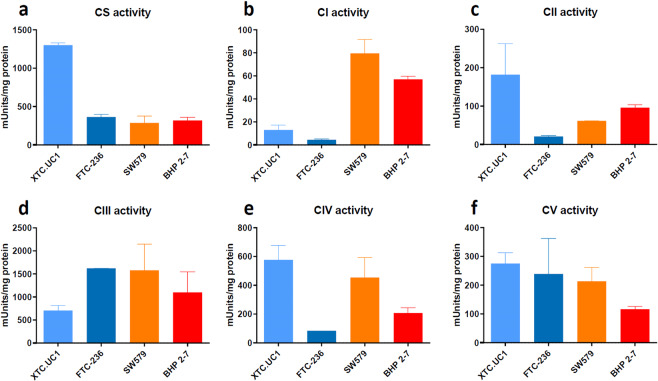
Absolute activities of citrate synthase and OXPHOS complexes in XTC.UC1, FTC-236, SW579 and BHP2-7; (**a**). citrate synthase (CS); (**b**). CI; (**c**). CII; (**d**). CIII; (**e**). CIV; (**f**). CV. Values are given as mean ± SEM. Measurements were repeated twice and each measurement includes two replicates. Note the high CS activity in XTC.UC1 and the significant loss of CI activity in both XTC.UC1 and FTC-236. CII, CIII, CIV and CV activities are more variable between the four cell lines.

Citrate synthase activity is confined to the mitochondrial matrix and therefore can be used as a marker for mitochondrial mass. Consistent with literature reports, higher levels of citrate synthase activity were observed in XTC.UC1 compared to other cell lines (Fig. 1a), a finding consistent with hyper proliferation of mitochondria. Activities of the OXPHOS enzyme complexes CI and CIII were also low in XTC.UC1 (Fig. 1b,d), which might be related to reported mtDNA mutations, as XTC.UC1 cells carry pathogenic variants in ND1 and cytochrome b. In addition, the FTC-236 cell line showed diminished CI, complex II (CII) and complex IV (CIV) activities compared to SW579 and BHP 2–7 (Fig. 1b–e). Loss of CI can cause a secondary reduction of CIII and/or complex IV (CIV). The reduction of CII activity can’t be explained via this mechanism since this complex is not part of the respirasome^31^. The high CI activity evident in SW579 (Fig. 1b) argues against the pathogenic relevance of the ND5 m.12634 A > G: p.100I > V variant (see Supplementary Table S1). In summary, XTC.UC1 and FTC-236 show diminished OXPHOS enzyme activities, while both SW579 and BHP 2–7 show substantial OXPHOS enzyme activities (Fig. 1).

### Glutathione levels and ROS in TCCLs

**Figure 2:**
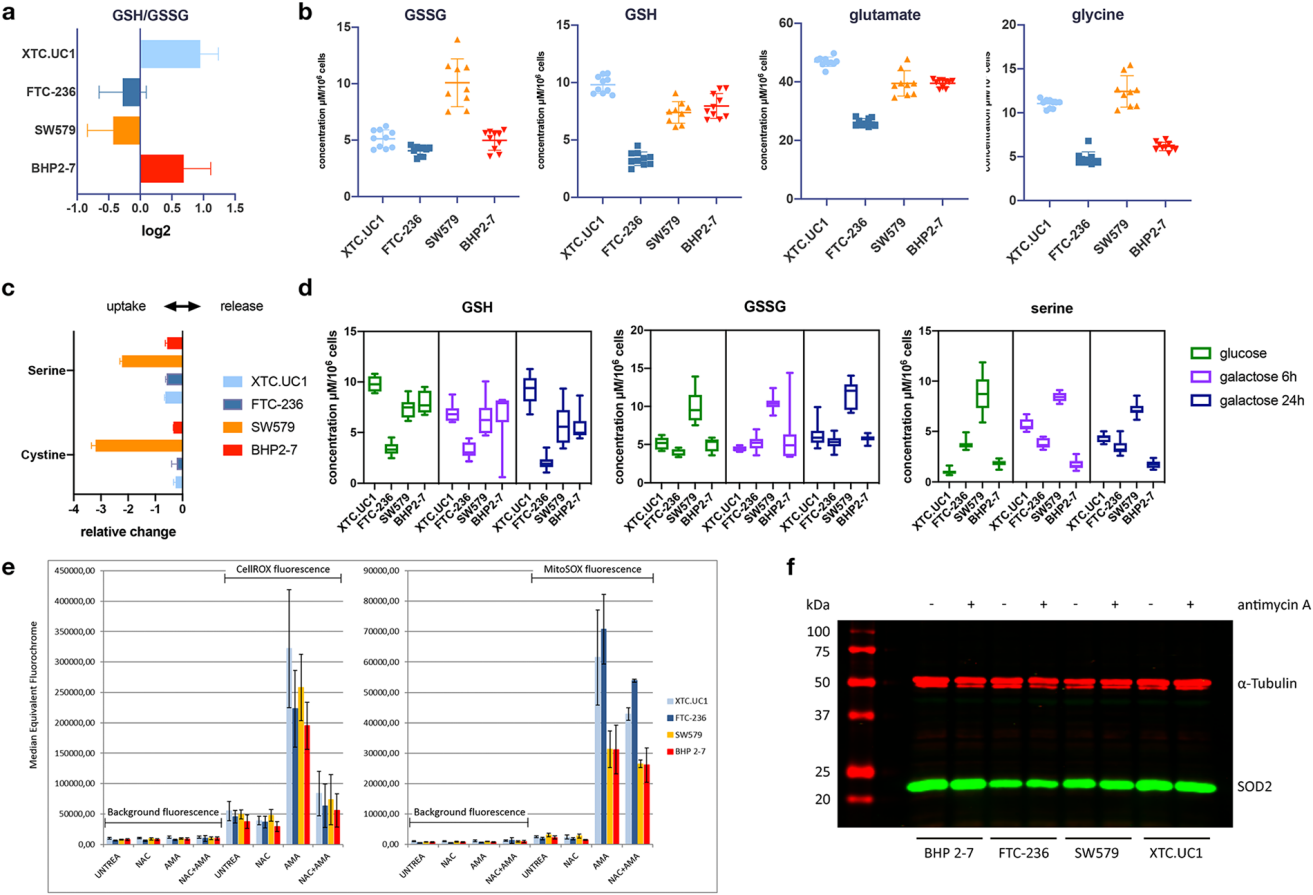
Intracellular levels of glutathione and ROS in the TCCLs, XTC.UC1, FTC-236, SW579 and BHP2-7; (**a**) ratio of reduced to oxidized glutathione, GSH/GSSG; (**b**) intracellular concentrations of GSH, GSSG and the glutathione precursors, glutamate and glycine; (**c**). uptake of glutathione precursors cysteine and serine from the culture medium (expressed as relative change, i.e. μmol/h/10^6^ cells of each metabolite in each cell line relative to the mean); (**d**). intracellular levels of GSH, GSSG and serine under normal culture condition (glucose) and under glucose to galactose exchange for 6 and 24 h; (**e**). Flow cytometric analysis of ROS: left panel, hydroxyl radicals detected by CellROX, right panel superoxide radicals detected by MitoSOX. Background fluorescence (left half each panel) and after incubation with CellROX or MitoSOX, respectively (right half). Fluorescence was analysed after cells were either untreated, NAC-treated, AMA-treated or AMA + NAC-treated. f. SOD2 immunoblotting (green signal). α-Tubulin was used as internal reference (red signal). A short treatment with 100 µM AMA did not affect protein expression. Left lane, molecular weight markers. Top and bottom of the image were cropped. For full-length gel, see end Supplementary Information.

The reaction of ROS with reduced glutathione (GSH) leads to increases in oxidized glutathione (GSSG), so higher levels of intracellular GSSG generally indicate increased ROS production. We used quantitative NMR to measure the intracellular levels of GSH and GSSG. Our analysis showed only minimal differences in the ratio of GSH/GSSG in the four cell lines, with XTC.UC1 and BHP2–7 showing a positive ratio (more GSH) while FTC-236 and SW579 had more GSSG than GSH (Fig. 2a). It should be noted that SW579 culture medium contained a low amount of GSH (3.3 µM). It is noteworthy that the cell lines harbouring a NHG had the highest GSH (XTC.UC1) and the lowest combined levels of GSH and GSSG (FTC-236) among the four cell lines. GSH levels were mirrored by intracellular levels of glutamate and glycine (Fig. 2b), both of which are precursors in glutathione synthesis. A third precursor of GSH, cysteine, was not detected and we did not observe significant differences in cysteine uptake (Fig. 2c).

We also measured GSH/GSSG after challenging the cells by culturing in glucose-depleted medium for 6 and 24 h, as lack of glucose shifts dependency to OXPHOS in terms of energy needs. We found that both GSH and GSSG were only slightly decreased (Fig. 2d), and GSH levels in XTC.UC1 and BHP 2–7 were still higher than in SW579, despite the fact that the RPMI-1640 medium used to culture SW579 contains GSH. Next, we examined other possible sources of glutathione biosynthesis and observed that intracellular serine was significantly increased upon glucose depletion in XTC.UC1 cells alone, remaining at the same levels in the other cell lines (Fig. 2d).

Since levels of glutathione, a H_2_O_2_ scavenger, differ between the cell lines we then asked whether ROS production under normal culture conditions correlates with glutathione levels. We used quantitative flow cytometry to measure superoxide anion (O_2_^−^) under basal conditions and during inhibition of oxidation of the ETC component ubiquinol (CIII) using 100 µM antimycin A (AMA) for 2 hours (optimal concentration: see Supplementary Figs 2 and 3). We first ensured that concentrations of 100 µM AMA and lower did not alter the autofluorescence of cells, change scatter characteristics or induce cell death as determined by DAPI percentage.

Basal levels of O_2_^−^ and OH^.^ did not differ significantly between the cell lines (Fig. 2e). However, O_2_^−^ increased significantly after treatment with 100 µM AMA, especially in the NHG cell lines FTC-236 and XTC.UC1 (p < 0.0001 compared to BHP2–7 and SW579). AMA treatment also induced a strong increase of CellROX Green fluorescence at this concentration, a ROS indicator mainly responsive to OH^−^, an increase that could be significantly inhibited in all cell lines when simultaneously treated with N-acetyl cysteine (NAC), an H_2_O_2_ and OH^.^ scavenger. The signal amplitude of MitoSOX Red, which mainly reacts with the O_2_^−^ radical, was only slightly reduced by NAC.

We then measured the expression of SOD2, the enzyme that catalyses the conversion of O_2_^−^ to H_2_O_2_ and O_2_. BHP2-7 and XTC.UC1 showed relatively higher expression of SOD2 compared to SW579 and FTC-236, the latter showing the lowest level (Fig. 2f). In conclusion, of the four cell lines XTC.UC1 was the most prominent, in that we found significant increases of the GSH precursor serine following culture in glucose-free medium, of O_2_^−^ after treatment with 100 µM AMA, and higher expression of SOD2.

### Metabolomic analysis of NHG and non-NHG TCCLs

**Figure 3:**
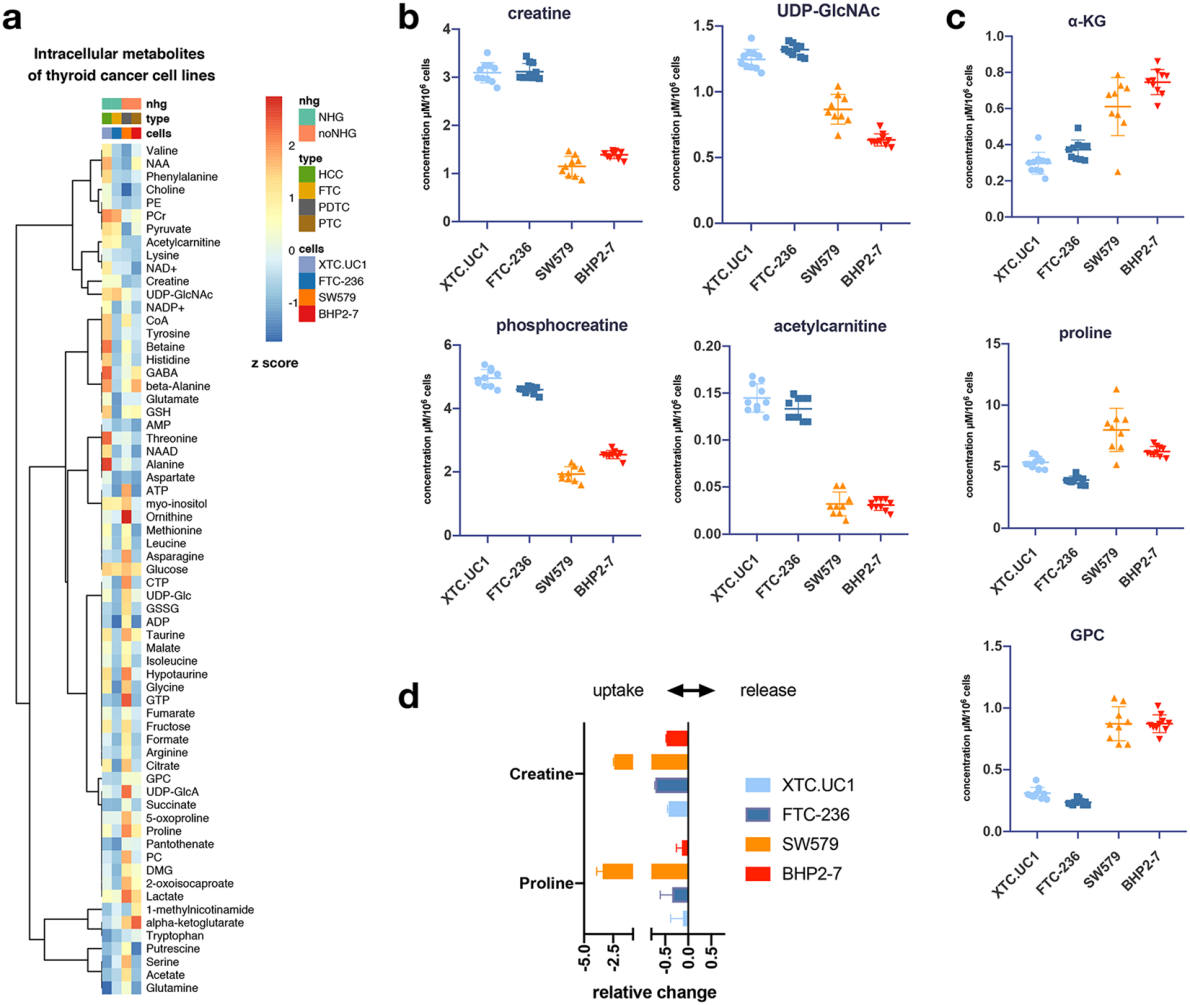
Metabolomics analysis of XTC.UC1, FTC-236, SW579 and BHP2-7; (**a**). heatmap of intracellular metabolites expressed as the z-scores of the mean quantified concentrations from n = 10 biological replicates per TCCL (for SW579, n = 9); (**b,c**). significant differences between TCCLs with and without a NHG; (**d**). uptake of the culture medium nutrients creatine and proline by the TCCLs (expressed as relative change, i.e. μmol/h/10^6^ cells of each metabolite in each cell line divided by the mean of the same metabolite).

We further analysed the four TCCLs using targeted measurement of intracellular and extracellular metabolites. Overall, each TCCL exhibited unique metabolic characteristics (Fig. 3a, Supplementary Figs. S4 and S8 and Supplementary Tables S2 and S3). Searching for common features between the two NHG TCCLs, we found that the intracellular levels of UDP-*N*-acetylglucosamine (UDP-GlcNAc), creatine, phosphocreatine (PCr) and acetylcarnitine were similar in XTC.UC1 and FTC-236 and significantly (p < 0.05) higher compared to the non-NHG SW579 and BHP 2-7 cell lines (Fig. 3b, Table 1). Furthermore, sn-glycero-3-phosphocholine (GPC), proline and α-ketoglutarate (α-KG) were also similar in NHG TCCLs and were lower overall compared to non-NHG TCCLs. (Fig. 3c, and Supplementary Table S4). Of these metabolites, only creatine and proline were provided in the culture medium and we observed a slightly higher uptake of proline by XTC.UC1 and SW579 and higher uptake of creatine by XTC.UC1 (Fig. 3d). However, small differences in uptake rates could not explain the large differences in intracellular pools in both NHG cell lines. A total of 50 metabolites were found to differ significantly between the two NHG cell lines XTC.UC1 and FTC-236 (adjusted p < 0.05, Supplementary Table S4). Similarly, 49 metabolites differed significantly between the two non-NHG cell lines, SW579 and BHP 2–7 (Supplementary Table S4).

**Table 1:** Concentration (µM/10^6^ cellls ± sd) of intracellular metabolites that exhibited similar levels in NHG vs no NHG showing TC cells.

metabolite	XTC.UC1		FTC-236		SW579		BHP 2-7		NHG/no NHG	
	mean	±sd	mean	±sd	mean	±sd	mean	±sd	fc	p
UDP-GlcNAc	1.246	0.077	1.321	0.045	0.867	0.113	0.634	0.046	0.786	1.06E-06
Acetylcarnitine	0.145	0.015	0.133	0.012	0.032	0.013	0.031	0.006	2.156	1.06E-06
Creatine	3.147	0.295	3.112	0.175	1.137	0.189	1.390	0.080	1.301	1.06E-06
Phosphocreatine	4.963	0.277	4.624	0.123	1.936	0.238	2.542	0.129	1.088	1.83E-09
alpha-ketoglutarate	0.273	0.041	0.378	0.057	0.611	0.161	0.766	0.074	−1.090	8.76E-06
GPC	0.366	0.044	0.217	0.022	0.723	0.091	0.885	0.049	−1.471	1.06E-06
Proline	5.346	0.478	3.905	0.323	7.984	1.760	6.225	0.411	−0.610	1.06E-06
Lactate	46.765	7.598	41.138	18.029	89.784	33.420	68.557	23.001	−0.839	8.41E-05

### NHG cell lines have lower glycolysis and favour synthesis of UDP-GlcNAc

**Figure 4:**
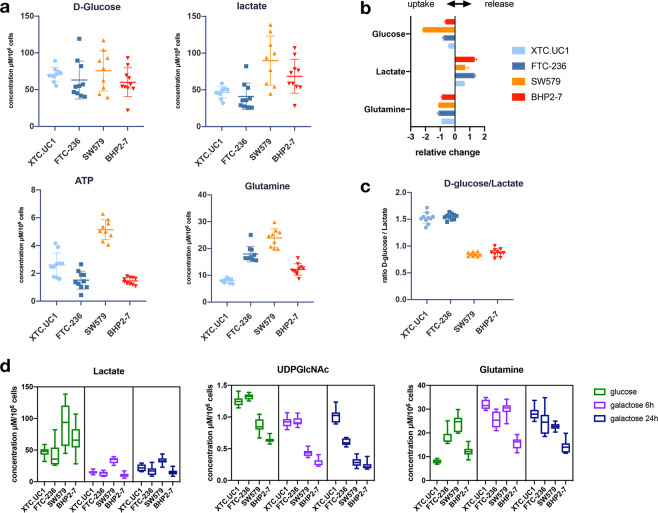
Uptake and metabolism of glucose and glutamine in XTC.UC1, FTC-236, SW579 and BHP2-7; (**a**). intracellular levels of D-glucose, lactate, glutamine and ATP; (**b**). uptake and release of D-glucose, lactate and glutamine (expressed as relative change, i.e. μmol/h/10^6^ cells of each metabolite in each cell line divided by the mean of the same metabolite); (**c**). ratios of intracellular D-glucose to intracellular lactate for each cell line; (**d**). intracellular levels of D-glucose, lactate and glutamine in normal culture medium and under glucose exchange by galactose for 6 and 24 h.

Following up on the observation that both TCCLs with a NHG produced higher amounts of UDP-GlcNAc, a product of the hexosamine pathway, we compared the glycolytic activity of all cells. We did not find any differences in intracellular pools of D-glucose (Fig. 4a) and uptake from the medium was also similar for all cell lines (Fig. 4b). However, we observed significantly lower intracellular lactate levels, as well as very little release to the medium by the NHG cell lines and especially by XTC.UC1, despite equal pools of glucose. Furthermore, the glucose to lactate ratio was higher in cell lines with a NHG (Fig. 4c). In order to further explore this finding, we measured the levels of the same metabolites after culturing cells in glucose-depleted medium for 6 and 24 h. As expected, following glucose depletion the intracellular levels of lactate were significantly reduced in all cell lines (Fig. 4d). UDP-GlcNAc intracellular pools also declined. In XTC.UC1 the level of lactate decreased by 6 h of glucose deprivation without a further reduction at the 24 h time point. Based on these observations, we suggest that NHG cell lines may utilize a significant fraction of glucose to synthesize the glycosylation substrate UDP-GlcNAc (Fig. 4d).

Glucose consumption was similar in NHG cells, as was the consumption of glutamine (Fig. 4b). However, despite a similar glutamine uptake, XTC.UC1 exhibited the lowest intracellular levels among all cell lines (Fig. 4a), suggesting that glutamine might be rapidly consumed in the synthesis of GSH and UDP-GlcNAc. After 6 hours of glucose depletion the average glutamine levels increased in all cell lines (albeit not to the same degree), suggesting a switch to greater glutamine uptake to compensate for the lack of carbon from glucose (Fig. 4d). Interestingly, XTC.UC1 maintained the intracellular pool of UDP-GlcNAc in the glucose-free medium, which further supports the hypothesis that glutamine contributes to the synthesis of UDP-GlcNAc in XTC.UC1 cells. Furthermore, glutaminolysis might also serve as an additional source for the increased glutathione synthesis seen in XTC.UC1.

### XTC.UC1 favours the *de novo* synthesis of aspartate

**Figure 5:**
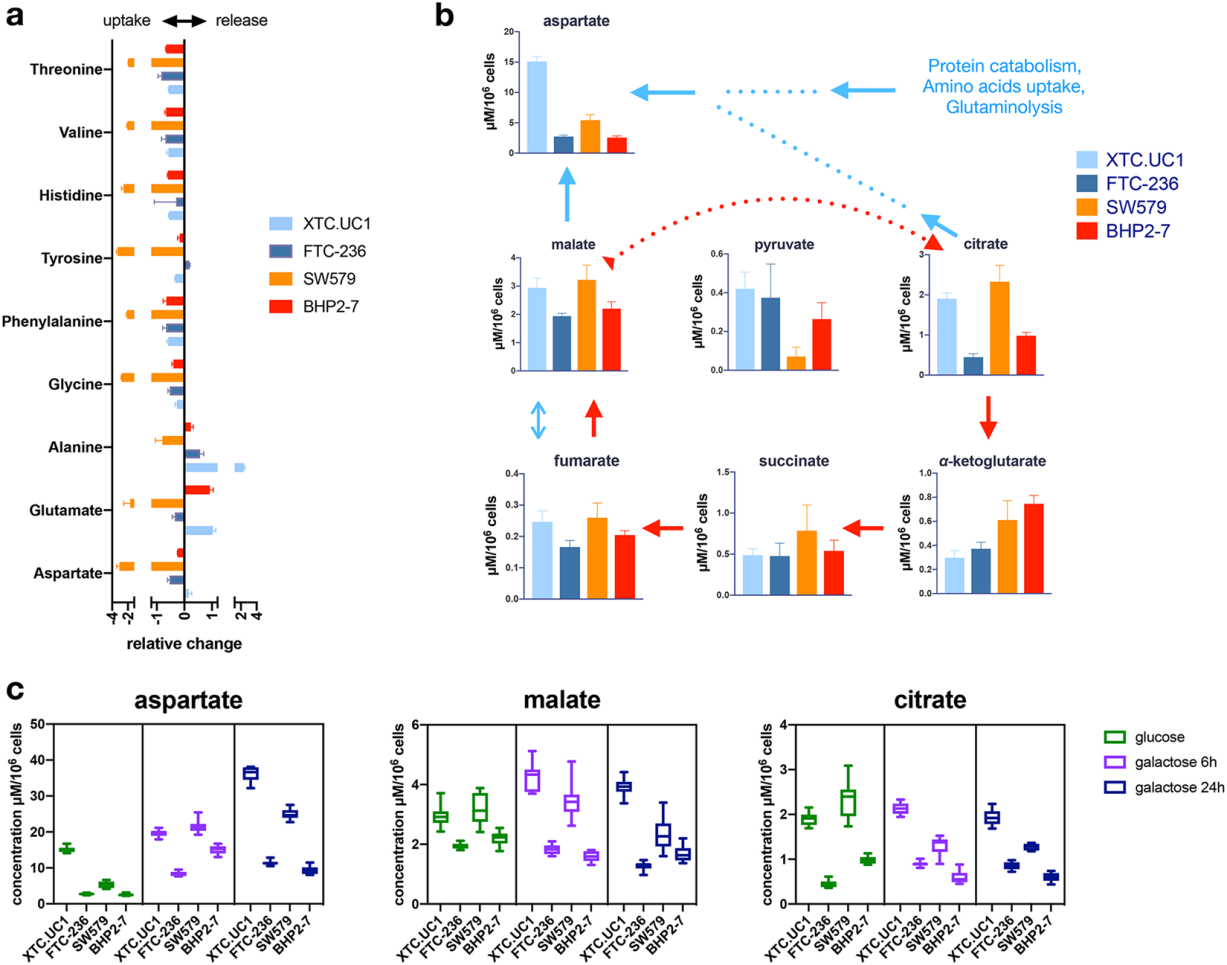
Central carbon metabolism in XTC.UC1, FTC-236, SW579 and BHP2-7 and *de novo* synthesis of aspartate; (**a**). consumption of nutrients and excretion of metabolic products (expressed as relative change, i.e. μmol/h/10^6^ cells of each metabolite in each cell line divided by the mean of the same metabolite); (**b**). schematic representation of the *de novo* synthesis of aspartate in the TCCLs and the quantified concentrations of TCA cycle intermediates and aspartate; (**c**). intracellular concentrations of aspartate and its precursors malate and citrate in normal culture medium and in glucose exchange by galactose medium for 6 and 24 h.

Based on the unique metabolic activity of XTC.UC1 with regard to glutamine utilization, we further studied its central energy metabolism. The most striking difference between XTC.UC1 and the other cells lines was the high intracellular pool of aspartate (XTC.UC1: 15.10 +/− 0.77; FTC-236: 2.74 +/− 0.23; SW579: 5.43 +/− 0.92; BHP 2–7: 2.57 +/− 0.27 µM/10^6^ cells) (Fig. 5b). XTC.UC1 also exhibited higher intracellular pools of several other amino acids, including valine, phenylalanine, tyrosine, histidine, threonine, alanine, *β*-alanine and glycine (Fig. 3a and Supplementary Table S5), while consuming these amino acids from the medium at levels comparable to FTC-236 (Fig. 5a). In addition, we found relatively high fumarate, malate and citrate levels but lower *α*-KG and succinate levels in XTC.UC1, suggesting the possibility of a highly active malate-aspartate shuttle (Fig. 5b). Finally, we observed that XTC.UC1 secreted aspartate to the medium, something not observed in the other 3 cell lines (Fig. 5a).

Galactose treatment forces cells to rely on OXPHOS for their energy needs. It resulted in slightly increased citrate and aspartate levels in XTC.UC1 and FTC-236 after 6 hours (Fig. 5c and Supplementary Fig. S5). In contrast, citrate levels decreased significantly in SW579 and BHP 2–7 after 6 hours of galactose treatment, strongly effecting their aspartate/citrate ratios (16.61 +/− 2.81 µM/10^6^ cells and 25.61 +/− 5.59 µM/10^6^ cells, respectively), and these ratios were significantly higher than in XTC.UC1 (9.28 +/− 0.76 µM/10^6^ cells) and FTC-236 (9.25 +/− 0.59 µM/10^6^ cells). Collectively, these data indicate that XTC.UC1 is able to utilize multiple sources to produce high amounts of aspartate (Supplementary Tables S2 and S3), and that this activity is independent of both the extracellular availability of aspartate and glycolytic activity. Regarding the other 3 cell lines, we also observed increased aspartate production in glucose-free medium, albeit to a lesser extent compared to XTC.UC1.

## Discussion

Metabolic adaptation plays an important role in the development of cancers characterized by abundant malfunctioning mitochondria (oncocytic phenotype), including thyroid Hürthle cell carcinoma (HCC)^32^. HCCs typically show widespread loss of entire chromosomes, leading to a near-homozygous genome (NHG)^7–9,13,21^. The loss of entire chromosomes has been attributed to aberrant interactions with reactive oxygen species (ROS)^21^. Interestingly, recent findings showed that ROS vary with the cell cycle phase and reach their highest levels during mitosis^33^. Thus, in cells with malfunctioning mitochondria, e.g. oncocytic cells such as XTC.UC1, mitosis might lead to greater cellular stress, increased levels of ROS and subsequent ROS-induced aneuploidy by chromosomal missegregation^34,35^. This mechanism may be regulated via enhanced CHK2 phosphorylation, as seen in mitotic cells after AMA treatment^21^, which could in turn contribute to lagging chromosomes and missegregation^36^.

In this study we investigated the basal levels of superoxide (O_2_^−^) and hydroxyl radical (OH^−^) production in thyroid cancer cell lines with (XTC.UC1 and FTC-236) and without an NHG (SW579 and BHP 2–7), and studied the effects of inhibiting CIII using antimycin A. We also studied OXPHOS complexes in TCCLs by spectrophotometric activity measurements and used standard and glucose-depleted cultures to evaluate glycolytic activity, GSH synthesis and additional metabolic alterations.

Similar to earlier studies of cybrid models carrying homoplasmy and various degrees of heteroplasmy^37^, we found no difference in the basal levels of O_2_^−^ (as detected by MitoSOX) in the four TCCLs tested, despite differences in mtDNA variants^21^. This suggests that under standard culture conditions mitochondrial H_2_O_2_ is rapidly catabolized by enzymes such as catalase, glutathione peroxidase and peroxiredoxin 3^38^, and only a fraction is converted to OH^−^, as shown by the limited effect of NAC. Interestingly, efficient detoxification of ROS is a suggested mechanism in HCC^39^. When we stressed the cell lines with 100 µm AMA, the NHG cell lines XTC.UC1 and FTC-236 generated significantly more O_2_^−^ than BHP 2–7 and SW579. Simultaneously a strong increase of CellROX fluorescence was observed, which could be reduced significantly by NAC. This shows that AMA treatment drives redox homeostasis towards H_2_O_2_, which can be neutralized by NAC. By contrast, MitoSOX fluorescence (which reacts mainly with O_2_^−^) was left relatively unaffected by NAC, indicating that SOD2-mediated dismutation to H_2_O_2_ is likely favoured above scavenging by NAC in the presence of active SOD2^40^.

Superoxide is mainly produced at CI and CIII of the ETC during normal respiration^41^ and levels increase significantly during AMA treatment^42^. Our data indicate that the ETC in TCCLs is (partially) functional even in the presence of a disruptive and/or damaging mtDNA variants^21^ and CI impairment in XTC.UC1 and FTC-236. Furthermore, overexpression of CIII and CIV, as described by Baris *et al*., suggests a compensatory mechanism for the relative loss of electron flow^39^. A functional ETC in the presence of CI mtDNA disruptive/damaging alterations might also be explained by the formation of respiratory supercomplexes that partially compensate for an impaired CI activity, as found in XTC.UC1 and in FTC-236 and as demonstrated in Ndufs4^−/−^ mice^43^. This process might also play a role in HCC^44^ and in renal oncocytomas showing loss of CI activity^23,45^. The latter studies showed that, like XTC.UC1, renal oncocytomas produce high levels of glutathione as a defence against partial impairment of CI of the ETC. Furthermore, Gopal *et al*.^23^ found that cysteine and glycine, precursors of glutathione, were reduced in renal oncocytomas, suggesting that their utilization may be increased due to flux towards glutathione synthesis. We observed no difference in the uptake of cysteine between XTC.UC1 and FTC-236 (Fig. 2c) and this source of cysteine is also unable to explain the intracellular differences in glutathione. However, we found that the levels of taurine, a cysteine degradation product, tracked intracellular GSH (Supplementary Fig. S6).

Interestingly, metabolic reprogramming was identified in the skeletal muscle of the aforementioned Ndufs4^−/−^ mice, including increased N-acetylaspartate (NAA)^46^ and *de novo* synthesis of aspartate^47^, together with its precursors pyruvate, fumarate, malate and citrate, and comparable to what was found in XTC.UC1 cells. This might reflect cytosolic GOT1 activity in the context of an impaired CI^48^, which partly counterbalances loss of mitochondrial aspartate synthesis^49^. The use of α-KG might indicate direct scavenging and the utilization of reductive carboxylation of α-KG by XTC.UC1 and FTC-236 to support growth compromised by defective mitochondria^50,51^. In addition, all TCCLs exhibited increased aspartate synthesis (Fig. 5c) when cultured under glucose deprivation. This might be due to low concentrations of pyruvate added to the galactose medium, essential to cells lacking a functional CI^49,52^.

Both XTC.UC1 and FTC-236 showed high levels of acetylcarnitine, which plays a key role during β-oxidation of fatty acids to generate ATP^53^. XTC.UC1^48^ and FTC-236 are CI compromised, thus β-oxidation might be an alternative source of ATP in these cells. Furthermore, acetylcarnitine supplementation of aging rats contributes to antioxidant defences, and to mitochondrial biogenesis and dynamics regulated by PGC-1α and PGC-1β^54,55^. Interestingly, PGC-1-related coactivator (PRC) and the NRF1 and TFAM transcription factors are upregulated in HCC^56^, highly comparable to the observations in acetylcarnitine-supplemented aging rats. The downstream effects of PRC were confirmed in XTC.UC1^10,11^. Thus, the high levels of acetylcarnitine in XTC.UC1 and FTC-236 might be a response to oxidative stress^55,57^, and the poor condition of the mitochondria in XTC.UC1 and FTC-236 might reflect accelerated aging^54,55^. Further support is found in the significantly lower levels of GSH, glutamate and glycine in FTC-236 (derived from a follicular thyroid cancer) compared to XTC.UC1 (HCC). This could indicate that the ROS scavenging capacity of FTC-236 is greatly reduced in comparison to XTC.UC1, BHP 2–7 and SW579, or that the GSH to GSSG turnover in FTC-236 is higher than in the other three cell lines. Yet another factor is the level of SOD2 expression, which is about two-fold lower in FTC-236 compared to XTC.UC1. Thus, in FTC-236 the conversion of O_2_^−^ to H_2_O_2_ might be rate-limiting due to lower SOD2 expression compared to XTC.UC1. Interestingly α-KG, likewise identified as an H_2_O_2_ scavenger^58,59^, was significantly lower in XTC.UC1 and FTC-236 compared to SW579 and BHP 2–7, which might indicate an active role in H_2_O_2_ scavenging in the first two cell lines. Despite the lack of excess O_2_^−^ and OH^−^ under standard culture conditions, in XTC.UC1 we observed not only higher GSH but also more GSH compared to its oxidized form GSSG, suggesting lower consumption due to a low proliferative activity and a balanced flux of GSH synthesis, as long as there is a constant supply of nutrients.

Many earlier studies reported an increased dependency of tumours on glycolysis. More recently, the importance of other metabolic pathways in tumorigenesis, tumour progression and survival has become clear^60,61^. We showed that XTC.UC1 and FTC-236 are less glycolytic and less OXPHOS competent compared to the BHP2-7 and SW579 cell lines. At first glance, FTC-236 appears to show classic Warburg-like characteristics due to low levels of TCA intermediates and low oxidative respiration. However, and similarly to XTC.UC1, FTC-236 showed lower levels of intracellular (Fig. 4a,d) lactate and very high levels of creatine/phosphocreatine and UDP-GlcNAc (Figs. 3b and 4d). Lower levels of lactate might reflect the reported diminished lactate dehydrogenase A expression in HCC^62^. This is in line with the increased levels of pyruvate in XTC.UC1 and FTC-236 (see Fig. 5b) compared to SW579 and BHP 2–7. Secondly, a fraction of the lactate can be utilized for lipid synthesis^63^. Furthermore, lactate has also been identified as a TCA cycle carbon source in non-small-cell lung cancers. This was most apparent in patients with an aggressive tumour showing a high ^18^fluorodeoxyglucose uptake^64^, as can be observed in HCC^65^. In this context it is noteworthy that both XTC.UC1 and FTC-236 has been derived from metastasis. Low levels of lactate might also contribute to the relative low proliferative activity of XTC.UC1 compared to the other TCCLs (Supplementary Table S3). Low proliferative activity is also seen in clinical HCC.

The high levels of creatine and phosphocreatine might point to an alternative route to ATP generation in XTC.UC1 and FTC-236 and direct ROS scavenging via creatine^66–69^, in line with a relatively quiescent glycolytic state^21,70^. Apparently, glucose is used to fuel other glycolytic pathways, such as the hexosamine pathway which supports UDP-GlcNAc synthesis (Supplementary Fig. S7). In XTC.UC1 specifically, the levels of UDP-GlcNAc are partially maintained even upon glucose depletion from the media, once again suggesting a flexibility of HCC to adapt its metabolism.

The important role of glutamine in the metabolism of XTC.UC1 is reflected in an approximately four-fold increase after 6 h in a glucose-depleted medium (see Fig. 4d). This may correlate with the observed increase of aspartate synthesis in XTC.UC1 after 24 hours of glucose depletion. However, this increase was less pronounced in FTC-236, SW579 and BHP 2–7. XTC.UC1 was also the only cell line showing a significant increase of intracellular serine, a major source for one-carbon metabolism (see Fig. 2d). Serine can feed the production of one-carbon units for use in nucleotide synthesis, methylation, providing ATP^71^ and associates with formate and alanine excretion, found at high levels in all four TCCLs. This underscores this pathway as an interesting target for inhibition when combined with decreased glycolytic ATP production.

To define common metabolic characteristics associated with NHGs in TC, we studied four distinct TCCLs with and without a NHG. We demonstrated that XTC.UC1 and FTC-236 are CI impaired, a phenomenon found earlier in HCC^22^, and which might correlate with a NHG in these tumours. Furthermore, our data points to a subtle balance between ROS generation and scavenging, directly via creatine and/or α-KG and/or conversion of ROS intermediates^72^. This might explain ROS-induced w-CIN in HCC and perhaps other rare cases of follicular thyroid cancer showing w-CIN^73^. Moreover, our data suggests phosphocreatine cycling and β-oxidation as alternative routes for generating ATP in XTC.UC1 and FTC-236, otherwise relatively quiescent glycolytic cell lines. Besides the pivotal role of acetylcarnitine in β-oxidation, the high levels found in the NHG cell lines XTC.UC1 and FTC-236 might provide protection against aged mitochondria. Importantly, we also found a profound metabolic switch of the HCC cell line XTC.UC1 towards the synthesis of aspartate. Bearing in mind the recent findings regarding the role of aspartate in cancer metabolism^47^, to the best of our knowledge our data shows, for the first time, that *novo* aspartate synthesis also plays a key role in HCC cells and might be considered a treatment target in this type of thyroid cancer.

## Supplementary information

Supplementary information.

Supplementary Tables.
