# Supplementary material for: Metabolic reprogramming related to whole-chromosome instability in models for Hürthle cell carcinoma
Source: Sci Rep. 2020 Jun 12;10:9578. doi: 10.1038/s41598-020-66599-1 (PMC7293353; doi:10.1038/s41598-020-66599-1)
Supplement: Supplementary file 1 — Supplementary information. [file 41598_2020_66599_MOESM1_ESM.pdf]

Supplementary Files

Metabolic reprogramming related to whole-chromosome instability in models for Hürthle cell carcinoma

Ruben D. Addie[1,2], Sarantos Kostidis[2], Willem E. Corver[1], Jan Oosting[1], Sepideh Aminzadeh-Gohari[3], René G. Feichtinger[3], Barbara Kofler[3], Mehtap Derya Aydemirli[1], Martin Giera[2], Hans Morreau[1]

Affiliations:

1. Department of Pathology, Leiden University Medical Center, Albinusdreef 2, 2333ZA, Leiden, The Netherlands
2. Center for Proteomics and Metabolomics, Leiden University Medical Center, Albinusdreef 2, 2333ZA, Leiden, The Netherlands
3. Research Program for Receptor Biochemistry and Tumor Metabolism, Department of Pediatrics, University Hospital of the Paracelsus Medical University, Müllner Hauptstraße 48, 5020 Salzburg, Austria

[*] Corresponding author: Willem E. Corver, PhD, Dept. of Pathology, Leiden University Medical Center, P.O. Box 9600, L1-Q, 2300 RC, Leiden, The Netherlands, w.e.corver@lumc.nl

Supplementary Table S1. Cell line characteristics (see reference 21)

Cell line	Gender	Age	Localization	Origin	NHG	DI	Complex	MT-gene	Mutation
**XTC.UC1**	f	63	soft tissue breast	HCC	Y	1.10	I / III	*ND1* / *CYTB*	m.3571dupC / m.15557 G>A: p.271E>K
**FTC-236**	m	42	neck lymph node	FTC	Y	1.22	I	*ND6*	m.14198 G>A: p.159T>I
**BHP 2-7**	f	u.k.	primary	PTC	N	1.07	-	variants	-
**SW579**	m	59	primary	FTC	N	1.58	I	*ND5*	m.12634 A>G: p.100I>V

u.k. = unknown

FTC = follicular thyroid cancer

HCC = Hürthle cell carcinoma

PTC = papillary thyroid cancer

NHG = near-homozygous genome (Yes or No)

DI = DNA index

MT-gene = mitochondrial gene

Supplementary Figures and Legends

Supplementary Figure S1

Staining scheme for semi-quantitative analysis of superoxide and hydroxyl radical in TCCLs by flow cytometry.

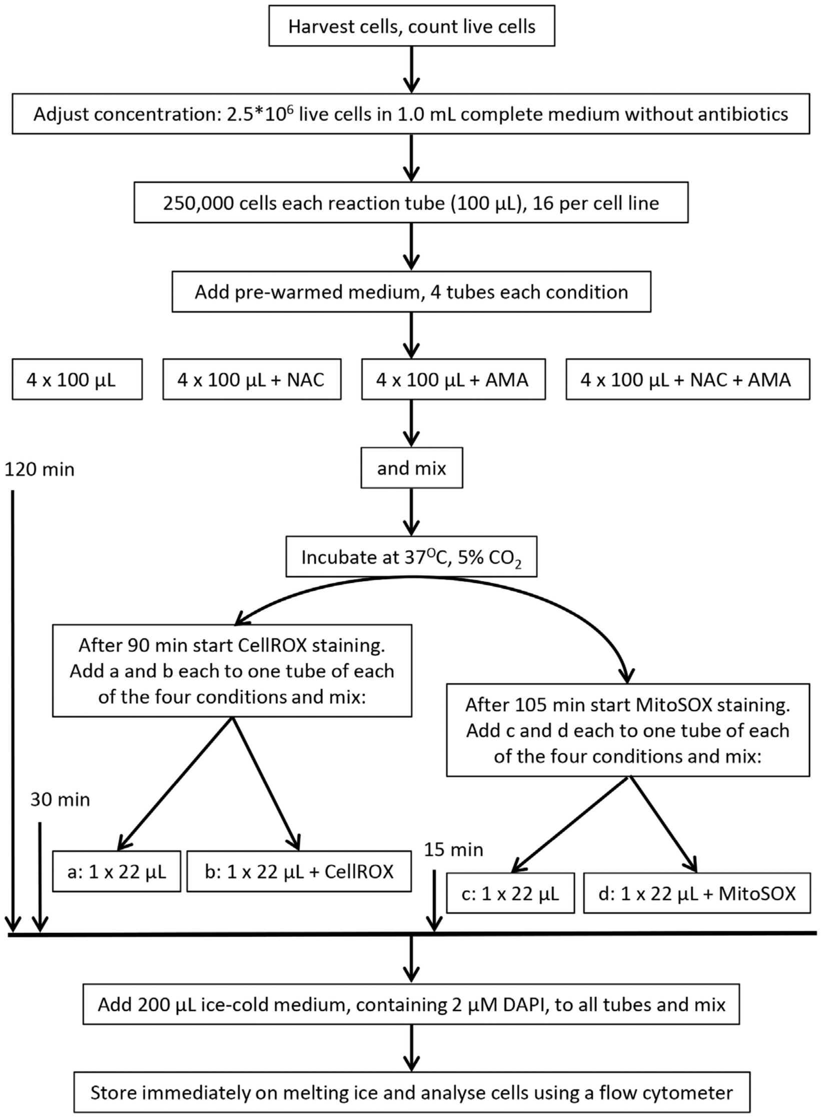

Supplementary Figure S2

Cell health and cell death after challenging TCCLs with different concentrations of AMA for 2 hours.

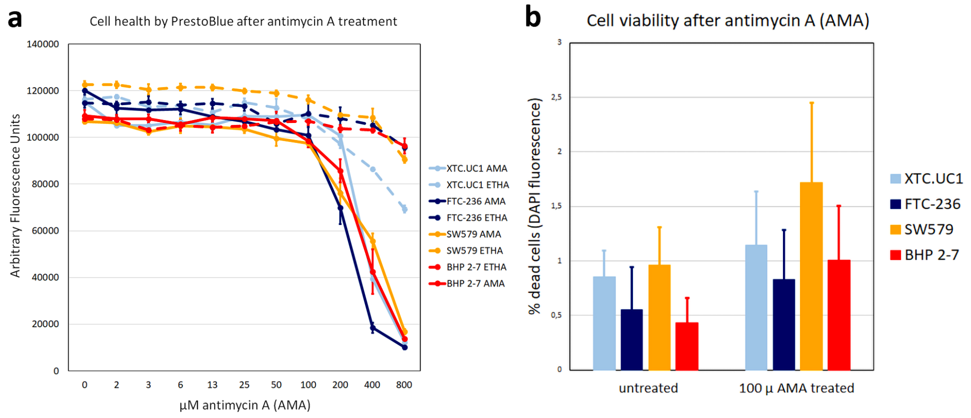

a. After 2 hours of incubation (37^{0}C, 5% CO_2) with different concentrations of AMA (range 1 – 800 µM) the medium was replaced by 100 µL fresh complete DMEM-F12 (XTC.UC1, FTC-236 and BHP 2-7) or RPMI1640 medium (SW579) supplemented with PrestoBlue according to the manufacturer instructions (Invitrogen, Thermo Fisher Scientific). After 2 hours fluorescence was measured using a Wallac Victor V, (PerkinElmer) plate reader at a wavelength of 560/10 nm and 590/10 nm, excitation and emission, respectively. Each condition was measured in fourfold. The entire experiment was repeated once with highly comparable outcomes and trends. b. AMA at 100 µM proved to be optimal. At this concentration the percentage cell death was determined by flow cytometry using DAPI fluorescence (dead and damaged cells) as a readout. After treatment with 100 µM AMA the percentage of dead cells increased slightly but not significantly.

Supplementary Figure S3

Gating strategy and fluorescence analysis of ROS by flow cytometry.

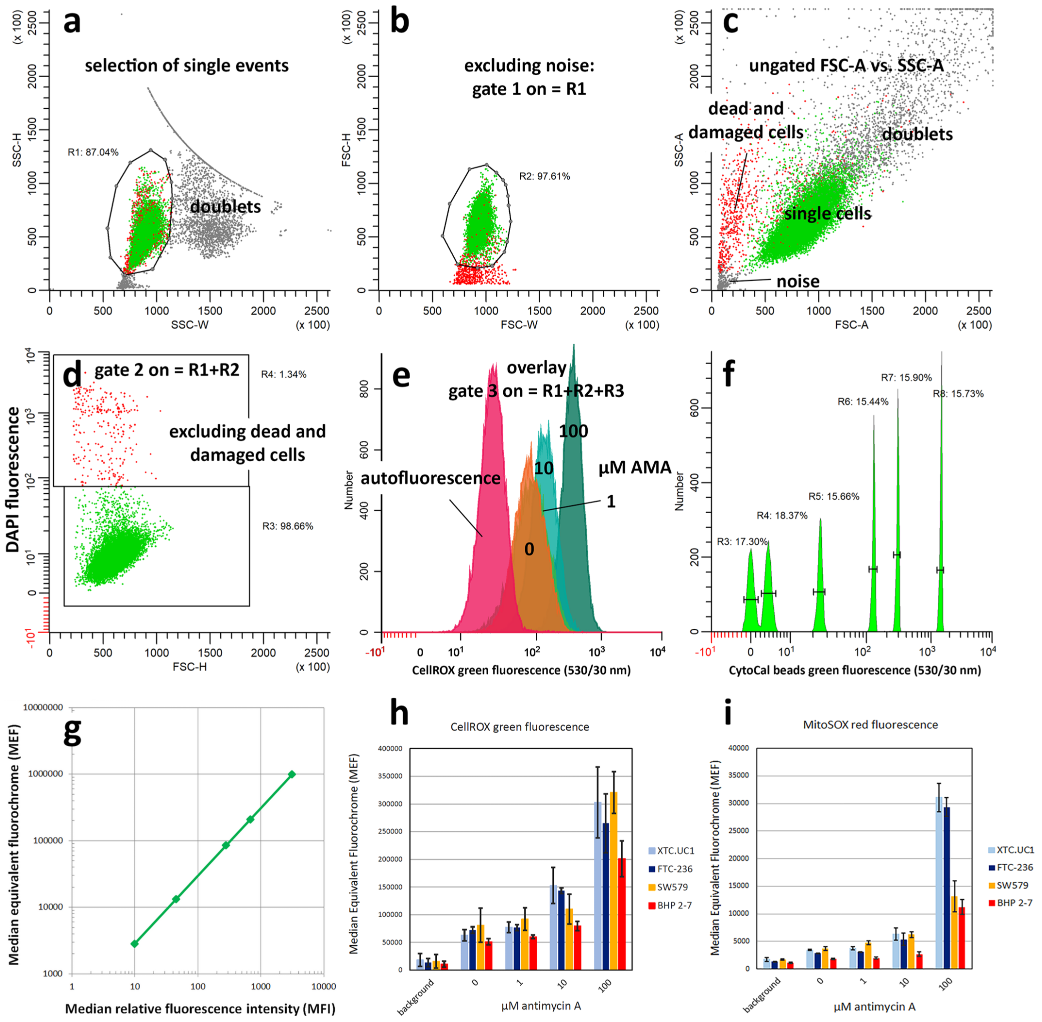

Thyroid cancer cells were treated and labelled according to the staining scheme (Supplementary Figure S1) and analysed by flow cytometry. BHP 2-7 cells stained with CellROX green are shown as a representative example.

a. Selection of single events using the side scatter width (SSC-W) vs. side scatter height (SSC-H) dot plot. b. Gate 1 active. Cellular fragments were further excluded using the forward scatter width (FSC-W) vs. forward scatter height (FSC-H) dot plot. c. Dot plot shows the ungated FSC-Area vs. SSC-Area. Note the different populations. d. Gate 2 active. Gating on live cells. e. Gate 3 active. Overlay of the autofluorescence and CellROX green fluorescence after 0, 1, 10 and 100 µM AMA, respectively. Note that the 1 µM AMA condition overlays almost completely with untreated BHP 2-7 cells. f. Fluorescence histogram of the six-peak CytoCal beads. g. Calibration line generated using the six-

peak CytoCal beads used to convert the median relative fluorescence intensities to median equivalent fluorochrome (MEF). h. MEF CellROX green fluorescence after challenging TCCLs with different concentrations of AMA for 2 hours (n = 3). Standard deviation is shown. i. Same as Figure h, but MitoSOX red fluorescence is shown (n = 3). Note that XTC.UC1 and FTC-236 produce significantly more superoxide than SW579 and BHP 2-7 using 100 µM AMA (see also Figure 2, an independent measurement).

Supplementary Figure S4

Overview of intracellular metabolites

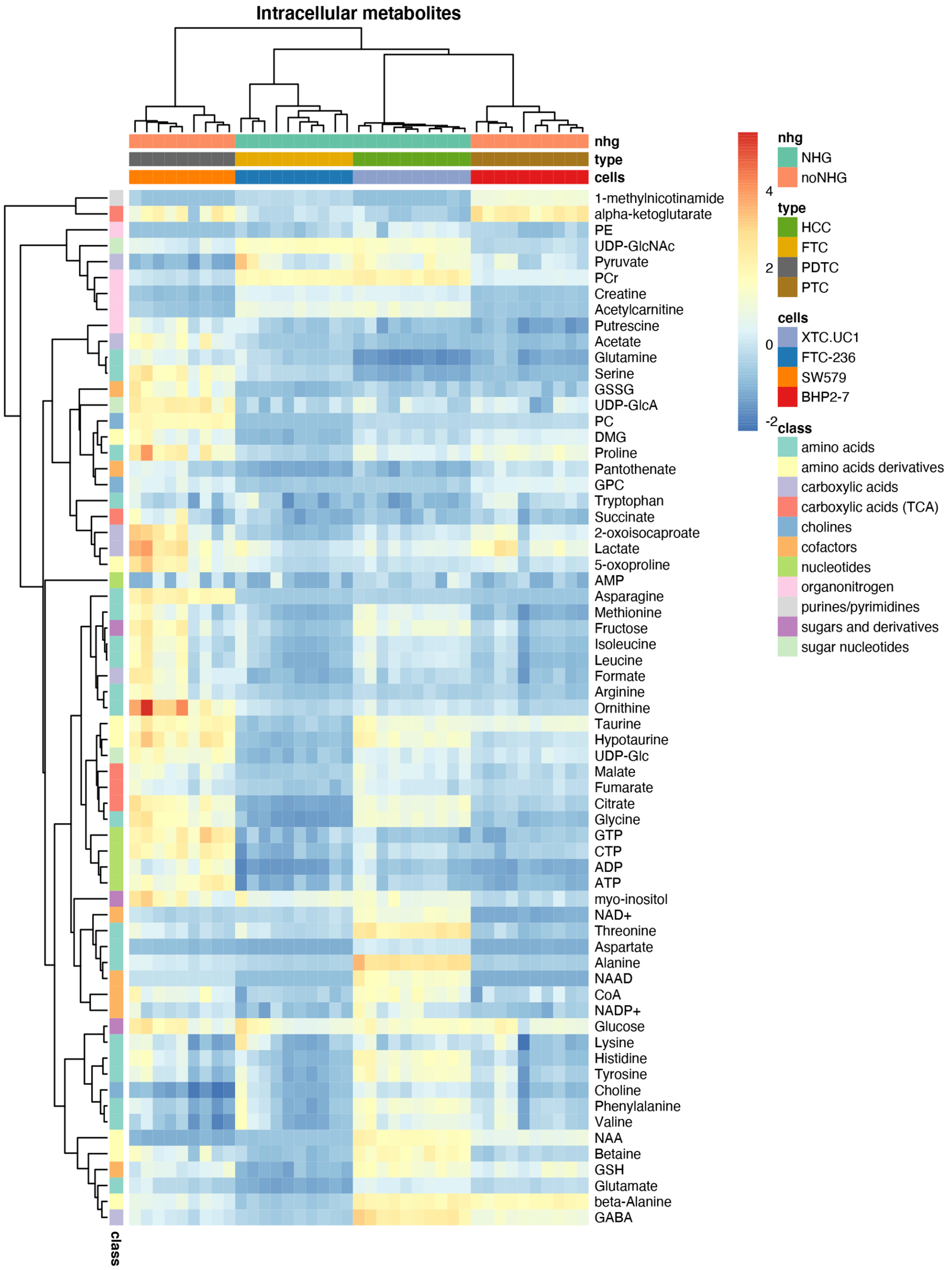

Overview of intracellular metabolites in XTC.UC1 (n = 10), FTC-236 (n = 10), SW579 (n = 9) and BHP2-7 (n = 10), expressed as z-scores of the quantified concentrations. Colour codes of upper horizontal bars show the group for each sample (e.g. NHG vs. no NHG) and the cell line tumour type (e.g. HCC, FTC or PTC). The vertical left sidebar shows the clustering of compounds into different chemical

classes. Overall, a very good intragroup was obtained with clear differences among the four tested cell lines.

Supplementary Figure S5

Intracellular levels of pyruvate, succinate and fumarate

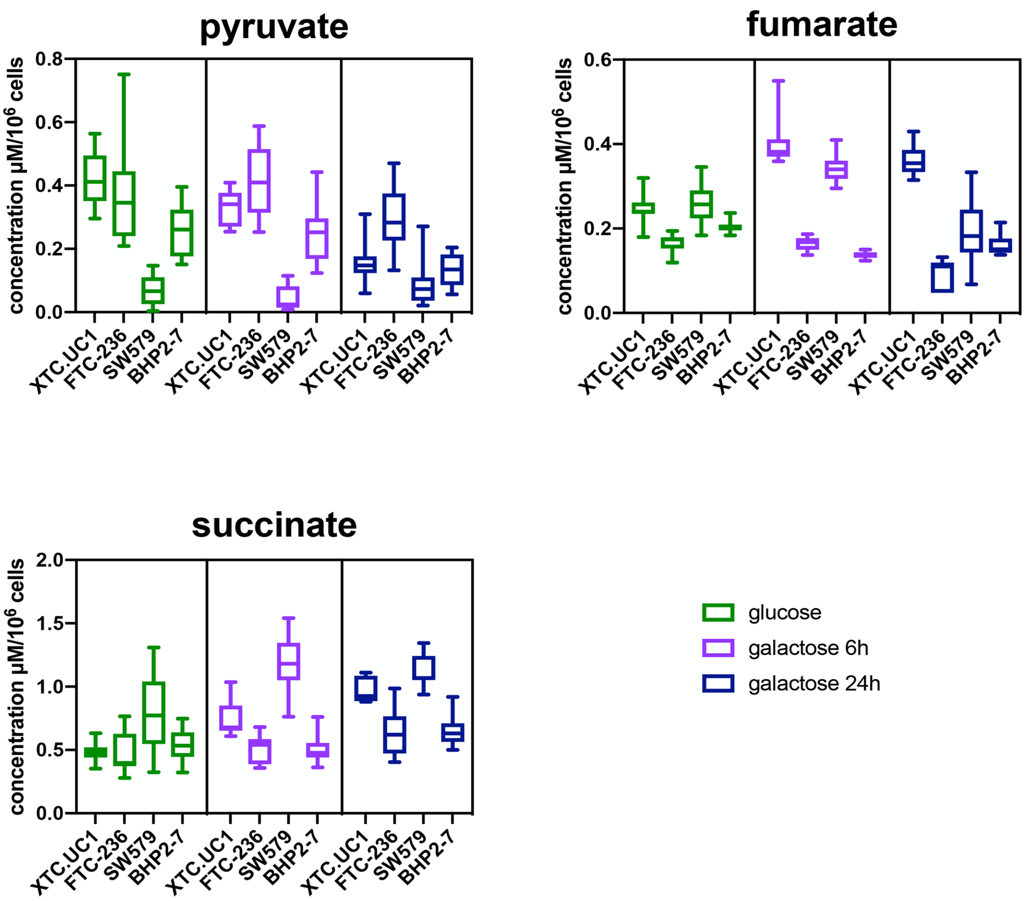

Intracellular levels of pyruvate, succinate and fumarate in XTC.UC1 (n = 10), FTC-236 (n = 10), SW579 (n = 9) and BHP2-7 (n = 10) cultured in standard medium and in medium with glucose exchanged by galactose for 6h and 24h.

Supplementary Figure S6

Intracellular levels of taurine

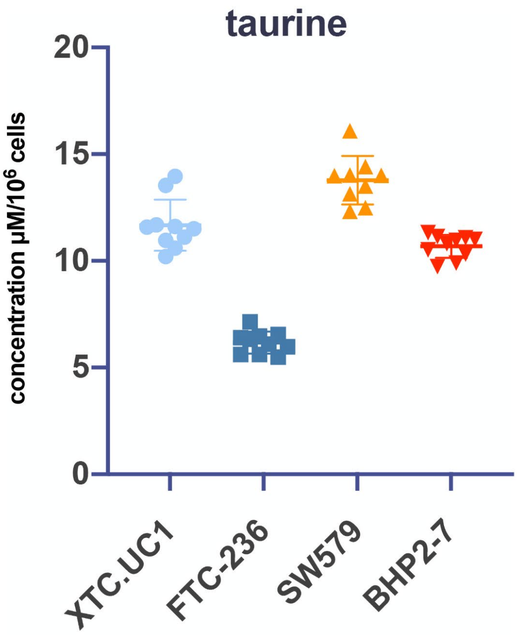

Intracellular levels of taurine in XTC.UC1 (n = 10), FTC-236 (n = 10), SW579 (n = 9) and BHP2-7 (n = 10).

Supplementary Figure S7

Intracellular levels of UDP-Glucose (UDP-Glc) and UDP-glucuronate (UDP-GlcA)

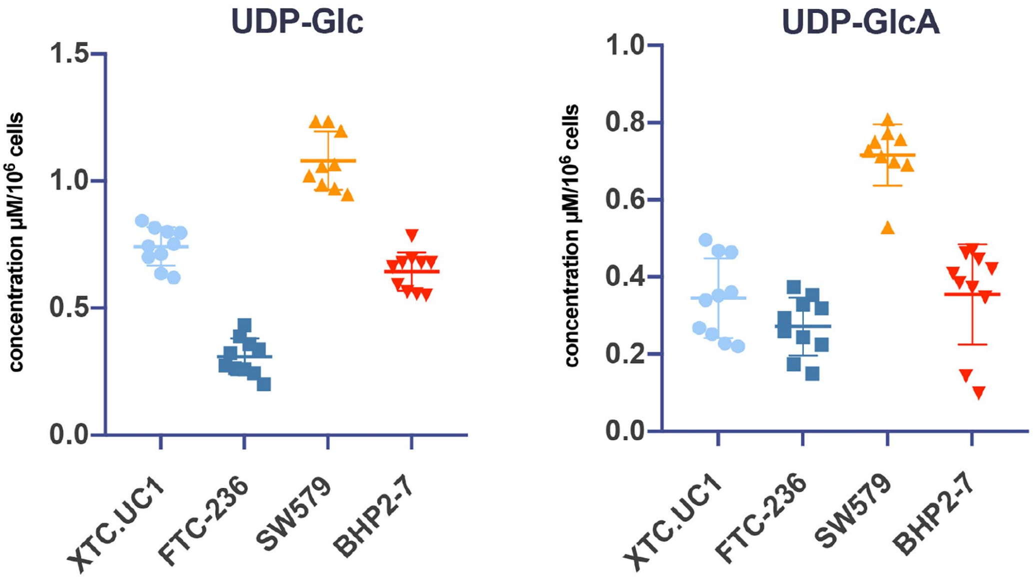

Intracellular levels of UDP-Glucose (UDP-Glc) and UDP-glucuronate (UDP-GlcA) in XTC.UC1 (n = 10), FTC-236 (n = 10), SW579 (n = 9) and BHP2-7 (n = 10).

Supplementary Figure S8

Dendrogram of hierarchical cluster analysis

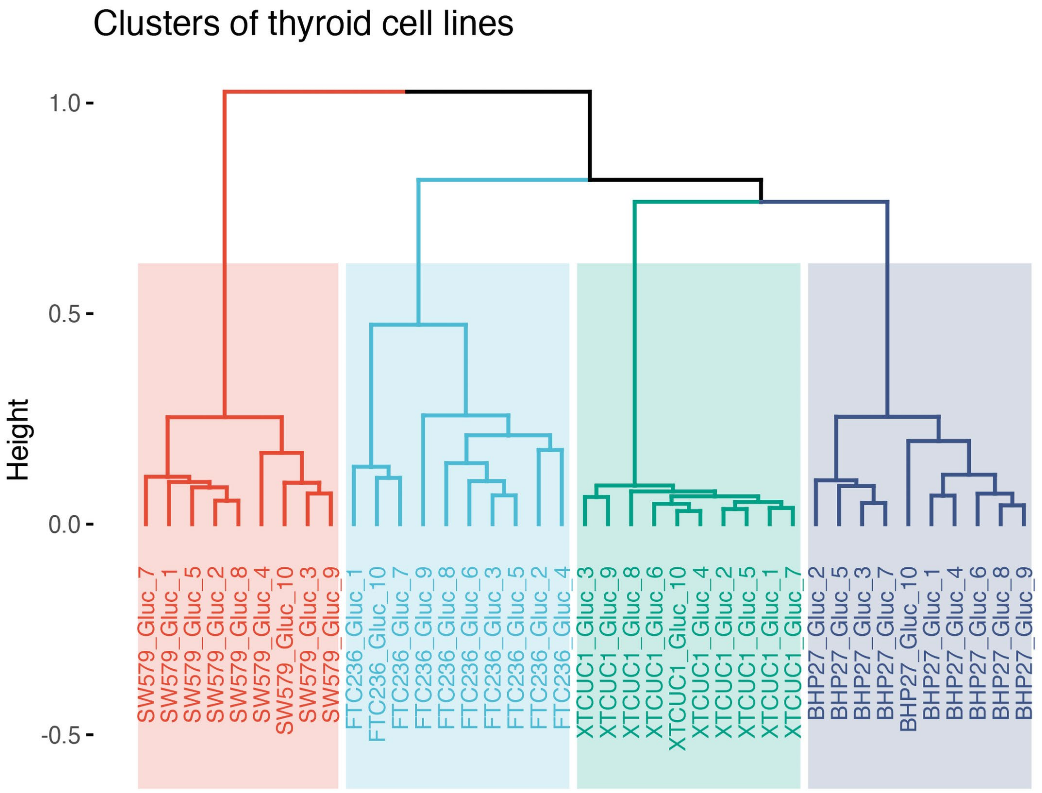

Dendrogram of hierarchical cluster analysis of the samples used in the study, based on their metabolomic data, showing that all samples per cell line form a unique cluster. Clustering was performed based on the average Spearman correlation between samples. Different colours are used for each cell line. Red: SW579, blue: FTC-236, green: XTC.UC1 and navy blue: BHP2-7.

Supplementary Figure S9

Full-length SOD2 (green signal) and α-tubulin blot (red signal)

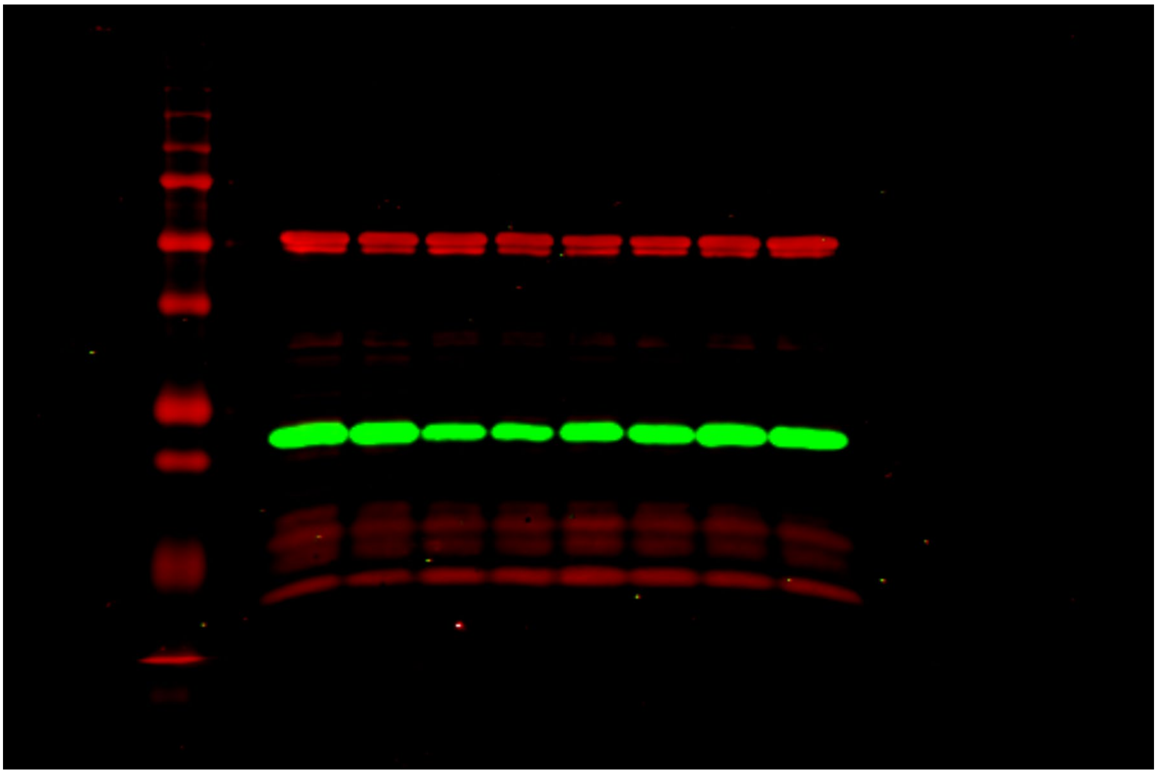

Left lane; size markers (red signal). For detailed description see Figure 2f and Materials and Methods; Western blotting. Relative imaging intensities: 1.5 and 3.0 for the fluorescent colour channels red (700) and green (800), respectively.
